# Understanding Ingroup Identification and Intergroup Threat Dynamics in Multiethnic Contexts

**DOI:** 10.5334/irsp.983

**Published:** 2026-03-23

**Authors:** Ena Uzelac, Margareta Jelić

**Affiliations:** 1 Department of Psychology, Faculty of Humanities and Social Sciences, University of Zagreb, HR

**Keywords:** ethnic identity, ethnonationalism, symbolic threat, intergroup anxiety, multiethnic contexts, majority-minority status

## Abstract

People’s tendency to perceive groups they do not belong to as potentially threatening, even when no actual threat exists, aligns with the fact that humans are social beings who highly identify with their groups. However, the perception of threat can severely damage social relationships, making it crucial to understand why and how it occurs in specific contexts. Hence, the aim of this study was to examine the longitudinal interplay between ethnic identity and ethnonationalism as different types of ingroup identification, along with various forms of intergroup threat. We also examined the moderating role of majority/minority group status and post-conflict/non-conflict multiethnic research contexts. The study was carried out at two time points, a year apart, and involved 852 adolescents (age range: 12–20; *M*_age_ = 15.69, *SD*_age_ = 1.77) from Croatia. The structural equation modeling (SEM) results suggest a reciprocal relationship between identity and the perception of threat. We found that experienced intergroup anxiety at T1 has a strong positive effect on ethnonationalism at T2. Furthermore, participants with a stronger ethnonationalism at T1 experienced higher levels of symbolic threat at T2, and a stronger perception of symbolic threat at T1 led to greater ethnic identification at T2. Contrary to our expectations, the relationships between variables operate in the same way across groups, regardless of group status and intergroup context. We discuss how this interplay between ingroup identification and intergroup threat affects the intergroup relations of adolescents in multiethnic contexts.

Prominent meta-theoretical approaches for studying intergroup dynamics are intergroup threat theory (ITT) (Stephan & Stephan 2000; Stephan & Renfro 2002) and social identity theory (SIT) (Tajfel 1978; Tajfel & Turner 1979), both of which belong to motivational approaches to explaining intergroup outcomes. However, the sources of motivation are somewhat different. While motivation for devaluing outgroup members according to ITT stems from the perception of threat to one’s own group, regardless of whether the actual threat is present or not (Stephan & Stephan 2000), the primary motivational force driving behavior according to SIT is the desire for positive social identity (Hornsey 2008). Nonetheless, both ITT and SIT emphasize that the strength of identification with a particular group plays a role in determining the intensity of the perceived threat and the reaction to it. Threat can manifest itself in various forms, including realistic and symbolic threat, as well as intergroup anxiety (Stephan, Renfro & Davis 2008). When a greater number of different groups share a certain territory over the years, studying the intricate relationship between social identities and perceptions of threat from those with whom group identity is not shared becomes particularly interesting. Precisely because their own social groups are so important for their identity, people are more prone to favoring their own group and even showing hostility toward other groups, especially in dangerous and uncertain times (Branscombe et al. 1999a; Chang, Krosch & Cikara 2016; Tajfel & Turner1979) and when there is a history of intergroup disagreements, conflicts, and misunderstandings (Stephan & Stephan 2018).

## Ingroup Identification and Perception of Intergroup Threat

Numerous studies have confirmed a positive correlation between ingroup identification and the perception of outgroup as a threat. A meta-analysis by Riek, Mania and Gaertner (2006) indicates that ingroup identification is a consistent predictor of both realistic and symbolic threat. However, whether stronger identification with one’s own group enhances the experience of realistic or symbolic intergroup threat, or intergroup anxiety, depends on the outgroup in question and the ingroup norms and narratives about the outgroup. In an effort to preserve their group’s identity or safety, those with a stronger ingroup identity will adhere to their group’s norms (usually by perceiving and expressing more threat). For instance, ingroup identification was shown to be a positive predictor of intergroup anxiety in a correlational study of attitudes of American whites toward blacks (Stephan et al. 2002), and it was later experimentally confirmed that the awareness that members of an outgroup provoke strong unpleasant emotional reactions in members of one’s own group increases anxiety among the participants themselves, which results in negative attitudes and discrimination against members of the outgroup (Stephan et al. 2005). Furthermore, Steele, Parker and Lickel (2015) in a study on American citizens’ attitudes toward Muslims found that the perception of realistic threat was stronger among participants who highly identify with their own group, while an experimental study by Pal (2021) demonstrated that highly identified adult Americans feel a greater symbolic than realistic threat from Muslims because the ethos at the time of the study was tied to political campaigns of “making America great again” and propaganda about preserving the American value system and traditional American identity. That highly identified individuals are prone to perceive symbolic threat is also confirmed by research conducted in a post-conflict context in Croatia, among ethnic majority Croats and post-conflict minority Serbs, both among adults (Löw Stanić 2014) and adolescents (Uzelac et al. 2022). Additionally, in the research by Uzelac et al. (2022), the group-identity-lens model (Verkuyten 2009) was confirmed, according to which high identification with one’s own (ethnic) group leads to a stronger perception of threat, which in turn leads to a stronger advocacy for assimilationist policies among members of the majority group, and a stronger advocacy for multiculturalism among members of minority groups. The differences in the prominence of different types of threats highlight the importance of contextual specifics in interpreting results, such as which groups are being studied and what the current political narrative is, as well as the timing of the research.

Ingroup attachment arises from the fundamental human need for belonging. However, when discussing ingroup identification in the context of ethnic groups, it is necessary to consider how an individual is connected to their ethnic group. Attachment to one’s people (ethnic identity) based on feelings of belonging and worthiness derived from this shared group identity should be distinguished from (ethno)nationalism, which presupposes a comparison and superiority of one’s ethnic group over others (Mummendey, Klink & Brown 2001; Schatz, Staub & Lavine 1999). Ethnonationalism can be understood as a negative pole of ingroup attachment that involves idealizing and glorifying one’s own ethnic group and mistrusting and derogating other ethnic groups (Blank & Schmidt 2003). Such a defensive and fragile identity is conceptually similar to national collective narcissism, which emphasizes that exceptionalism of one’s own group is insufficiently recognized by others. In contrast, ethnic identity represents a secure identity, characterized by a positive evaluation of one’s group that fulfils individual needs (Marchlewska et al. 2018). The relevance of distinguishing between these two forms of group identity, regardless of the underlying conceptualization, is confirmed by numerous studies that have shown that ethnonationalism (defensive, insecure, narcissistic identity), rather than secure ethnic identity, is harmful to the recovery of post-conflict communities, is associated with the intention to discriminate against members of the outgroup, and expression of overt prejudices against ethnic minorities (Bertin et al. 2022; Guerra et al. 2022; Jelić, Uzelac & Čorkalo Biruški 2020; Jelić, Čorkalo Biruški & Ajduković 2014; Penic, Elcheroth & Morselli 2017; Skokandić 2018). Also, its negative effects are achieved through the perceived symbolic threat and intergroup anxiety, as confirmed by research on prejudices of adolescents toward ethnic minorities in Croatia, prejudices of adolescents toward Roma in Serbia, Bulgaria, and Romania, and interethnic relations in America (Dimitrova et al. 2015; Jelić, Uzelac & Čorkalo Biruški 2020; Ljujic, Vedder & Dekker 2012; Skokandić 2018; Willis-Esqueda, Hazel Delgado & Pedroza 2017). The identity attached to one’s own ethnic group is most prominent in multiethnic contexts, and in Croatia, two broader categories of multiethnic contexts can be distinguished: the post-conflict context of the town Vukovar and its surroundings (Croatian-Serbian context) and non-conflict contexts, which include the Croatian-Italian context in several towns in Istria County, the Croatian-Hungarian context in the town of Osijek and its surroundings, and the Croatian-Czech context in the town of Daruvar and its surroundings.

## Multiethnic Contexts in Croatia

In Croatia, there are four ethnically heterogeneous contexts where minorities are large enough to have and practice their constitutional right to education in their mother tongue, each marked by different everyday dynamics between the majority Croats and the dominant minority group (see Appendix A for structure of participants by ethnicity). Although all these minority groups have preserved their ethnolinguistic vitality (Giles & Johnson 1987), i.e., their ability to maintain and protect their existence in time as a collective entity with a distinctive identity and language, the majority–minority relations in these contexts have developed under different historical and socio-economic factors. In each of the four intergroup contexts, our attention was directed toward the Croat majority group and one particular minority group (Serbs, Hungarians, Czechs, or Italians) with access to organized education in its native language (see Appendix A). All mentioned minority groups are equal before the law, and minority members in these contexts exercise their right to education in their mother tongue by attending either separate minority schools (usually in villages) or separate classes within Croatian schools taught exclusively in the specific minority language. However, the most prominent dimension of comparison and the key by which we have divided the contexts in this study is the history of conflicts among the groups. The Croatian-Serbian context stands out from the others due to the Homeland War of 1991–1995, whose consequences are still visible in ethnic divisions and difficult social reconstruction of the community (Čorkalo Biruški & Ajduković 2007, 2009; Jelić, Čorkalo Biruški & Ajduković 2021). Therefore, we dichotomised the context: the Croatian-Serbian context represents a post-conflict community, whereas the Croatian-Czech, Croatian-Hungarian, and Croatian-Italian contexts represent non-conflict communities because the conflicts with these groups were either very long ago or never existed.

## Relevance of the Present Study

Intergroup threat has mainly been researched in relation to immigrants and asylum seekers (Gregurović & Župarić-Iljić 2023; Kalebić Maglica, Švegar & Jovković 2018; Kiralj Lacković & Ajduković 2024; Kumpes 2018; Župarić-Iljić & Gregurović 2013), and it is not justifiable to equate immigrants with ethnic groups that have been living in Croatia for decades or centuries and have certain rights and a special status in society (Kymlicka 1995). Furthermore, the question is whether the perception of intergroup threat will be a relevant factor only in the post-conflict context, which is most similar to the environments where ITT was tested (e.g., Stephan, Ybarra & Rios Morrison 2009) or in non-conflict context as well. The authors of ITT emphasize the importance of testing the model in different contexts, with diverse groups, and considering the attitudes of both majority and minority group members. In this sense, the post-conflict and non-conflict contexts in Croatia offer an excellent opportunity to test the significance of different determinants of intergroup threat perception in real conditions, among majority and minority adolescents born and raised in these communities. Particularly important is the perspective of adolescents, which we are addressing in this study, who in our post-conflict context do not have the experience of growing up in a socially integrated multiethnic community, but were born after the war and have lived in an ethnically divided context since birth. Our study focuses on adolescents because of the profound influence that social contexts and interethnic interactions may have on this age group. The prevalence of negative sentiments toward outgroup members among adolescents can foster adversarial interethnic interactions during their schooling years, which may persist into early adulthood (Dimitrova et al. 2015).

Finally, drawing from the research on ethnic identity and ethnonationalism (Dimitrova et al. 2015; Jelić, Uzelac & Čorkalo Biruški 2020), as well as similar theoretical frameworks such as nationalism, or ethnic collective narcissism vs. identification (e.g., Bertin et al. 2022; Guerra et al. 2022; Spry & Hornsey 2007), we aim to distinguish between the effects of ethnic identity and (ethno)nationalism on perception of intergroup threats. This will allow us to better understand how each type of attachment to one’s group is connected to perception of the outgroup as a threat, and whether it is crucial to include different social identities as predictors of threat (and vice versa) in future studies of intergroup relations.

Hence, in this longitudinal study, we addressed the gaps in previous research by examining adolescents from diverse ethnic backgrounds, both from post-conflict and non-conflict contexts to understand the development of intergroup relations. We gathered data from a representative sample by including schools from both majority and minority groups in multiethnic contexts.

## Aim of the Study

The aim of this study was to examine the role of different forms of ingroup identification (ethnic identity and ethnonationalism) in predicting various forms of intergroup threat (realistic, symbolic, and intergroup anxiety), among adolescents from post-conflict and non-conflict multiethnic communities in Croatia. To establish the generalizability of longitudinal effects, we examined whether the presumed relationships were moderated by the majority–minority group status and the multiethnic research context.

Based on previous studies and social identity theoretical considerations, we expect ethnonationalism and ethnic identity to be positive predictors of various forms of intergroup threat, but different in strength, with ethnonationalism being a stronger predictor of threat than ethnic identity. Although we expected ingroup identification to predict threat perception, we acknowledge the possibility of reverse causality, as stated by ITT (Stephan & Stephan 2018). Namely, one of ITT’s main advantages is its adaptability to the research context, enabling the researcher to position and connect variables in ways that most closely reflect social reality. By additionally testing the possibility of reverse causal relationships, this study contributes to a better understanding of the associations among the variables. It builds on previous, predominantly cross-sectional research conducted in this context, using those findings as a starting point for the present study. Thus, while our hypotheses were grounded in theoretical reasoning, we also tested the reverse pathways, from threat perception to ingroup identification, to control for potential reverse causality and better clarify the directionality of these relationships.

Regarding the moderation of status, we expected that the longitudinal effects of ethnonationalism and ethnic identity on the perception of threat would be greater for members of ethnic minorities than for members of the ethnic majority. According to ITT, highly identified minorities, being lower in social power, may prioritize preserving their disadvantaged group, making them more sensitive to threats from the majority. Consequently, their focus is likely more on their group’s well-being and the power disparity compared to highly identified Croats. Finally, we anticipated that the longitudinal effects of ethnonationalism and ethnic identity on threat perception would be greater in a post-conflict multiethnic context compared to non-conflict multiethnic contexts.

## Method

### Participants and Procedure

The sample consisted of elementary and high school pupils belonging to ethnic minority groups in Croatia, who are educated in their mother tongue and script, as well as their peers, members of the ethnic majority—Croats, who are educated in the Croatian language and script in the same communities where schools in the minority languages are located or in their immediate vicinity.

The research was conducted at two time points, T1 and T2, in 24 elementary (8 majority, 7 minority, and 9 both majority and minority language of instruction) and 10 high schools (4 majority, 3 minority, and 3 both majority and minority language of instruction). We recruited pupils attending seventh grade of elementary and first three grades of high schools at T1 so that we can follow them in our panel study (see Appendix B for structure of participants with regard to the school and class). The first data collection took place during the winter and spring of 2017, with the second data collection occurring one year later. In both time points, the research team administered questionnaires during regular classes within one school hour (45 min) and instructed participants to generate a unique code, which allowed us to match their T1 and T2 data while keeping their identities anonymous. The researcher was accompanied by an assistant fluent in the students’ native language, if needed, to ensure clear understanding of the instructions and questionnaire items. The questionnaires intended for majority and minority pupils were identical, except that for majority pupils, the content of intergroup variables was directed toward members of the dominant minority in each context, while all minority students assessed Croats. Minorities predominantly completed the questionnaires in their native language, but they also had the option to complete them in Croatian.

Pupils were categorized as belonging to the ethnic minority or majority based on whether their classes were primarily taught in the minority or majority language. Although such categorization did not completely correspond with their ethnic identification, we argue that in multiethnic settings where mixed marriages are social reality, it is the choice of language of instruction that is a valid and consistent indicator of child’s ethnic affiliation. At T1, we collected data from 1,035 pupils who could be longitudinally followed, but in the final longitudinal sample, we had 852 pupils, of which 45.5% were male, and the age range at the first measurement point was from 12 to 20 years, with a mean age of 15.69 and a standard deviation of 1.77. Of these 852 pupils, 485 were Croats, and 367 were members of ethnic minorities, with altogether 360 from a post-conflict context, and 492 from non-conflict contexts.

### Measures

***Ethnic identity*** was assessed by five items adapted from Doosje, Ellemers and Spears (1995), which had been modified in previous research on majority–minority relations in post-conflict context of Vukovar, where the scale consistently showed a unifactorial structure (e.g., Čorkalo Biruški & Magoč 2009; Löw Stanić 2014). Sample items included “I am glad to be a member of my nation,” and “I feel strong ties with members of my nation.” The responses were indicated on a five-point scale ranging from strongly disagree (1) to strongly agree (5). Higher values indicated a stronger ethnic identity. The ethnic identity scale showed satisfactory reliability (k = 5, ωt at T1 = .92, α at T1 = .91, ωt at T2 = .94, α at T2 = .93).

***Ethnonationalism*** was assessed by three items used in Čorkalo and Kamenov (1998). The responses were indicated on a five-point scale ranging from strongly disagree (1) to strongly agree (5). Based on factor analysis, one item was excluded from the calculation of total results because it was equally saturated with both the factor of ethnic identity and the factor of ethnonationalism. Two remaining items were “My nation is better than other nations,” and “In all historical conflicts with other nations my nation was always right.”^1^ Higher values indicated more pronounced ethnonationalism. The measure of ethnonationalism showed satisfactory reliability (k = 2, α at T1 = .76, α at T2 = .80).

***Perceived intergroup threat*** was measured using a modified Intergroup Threat Scale (Čorkalo Biruški 2011) developed in the context of Vukovar based on the scale by Stephan and colleagues (2002), which encompasses the perception of both realistic and symbolic intergroup threat. Confirmatory factor analysis confirmed the two-factor structure. Perception of realistic and symbolic intergroup threat is measured by examining the degree of agreement with certain statements about the perception that the outgroup threatens the well-being of one’s own group in various ways. ***The perceived realistic threat scale*** consists of five items assessing the experience of discrimination against one’s own group, specifically the experience of endangered rights and opportunities. Sample items included “[Out-group members] in our town have more privileges (rights) than anywhere else,” and “Students whose language of instruction is [out-group] have more opportunities to participate in school competitions than students whose language of instruction is [in-group].” The responses were indicated on a five-point scale ranging from strongly disagree (1) to strongly agree (5). Higher values indicated more perceived intergroup realistic threat. ***The perceived symbolic threat scale*** consists of five items assessing the experience of threat due to differences in symbols, beliefs, and worldviews of the groups. Sample items included “My [out-group] peers do not respect the language of my nation,” and “My [out-group] peers should not overemphasize their national symbols and customs.” The responses were indicated on a four-point scale ranging from strongly disagree (1) to strongly agree (4), but were later recoded on a five-point scale to obtain directly comparable results and avoid potential issues with parameter estimation convergence when setting up linear structural models. Based on factor analysis, one item was excluded from the calculation of total results because it had large standardized residuals and was substantively applicable only to the post-conflict Croatian-Serbian context. Higher values indicated more perceived intergroup symbolic threat. The reliability of the perceived realistic intergroup threat scale proved satisfactory (k = 5, ωt at T1 = .85, α at T1 = .79, ωt at T2 = .88, α at T2 = .84), as did the scale of perceived symbolic intergroup threat (k = 4, ωt at T1 = .81, α at T1 = .83, ωt at T2 = .85, α at T2 = .82).

***Perceived intergroup anxiety*** was measured using a six-item scale modified from Stephan and Stephan (1985), asking participants how they would feel when interacting with members of the relevant outgroup, e.g., comfortable (reverse coded), and nervous. Participants responded on a five-point scale ranging from not at all (1) to extremely (5). Six indicators used to operationalize the latent factor of perceived intergroup anxiety were divided into three composite indicators, each comprising two items following the principles of creating balanced composite indicators (Weijters & Baumgarten 2022). This technique increased the parsimony of the measurement and structural model and reduced the impacts of various sources of potential measurement errors associated with each individual indicator (Little et al. 2002). The total score is expressed as the average of responses on composite indicators, and a higher score indicates more pronounced intergroup anxiety. The reliability of the perceived intergroup anxiety scale was satisfactory (k = 3, ωt at T1 = .86, α at T1 = .86, ωt at T2 = .88, α at T2 = .87).

***Socio-demographic characteristics*** such as age, gender, nationality, school, grade and residential status (i.e., place of living, household members, monthly income of the family) were examined.^2^

### Analytic Procedure

We analyzed the data using statistical software SPSS 29.0 (IBM Corp. 2022) and R 4.2.2 (R Core Team 2022).

The main analytical strategy was structural equation modeling (SEM) based on latent constructs. We assessed model fit using established absolute and incremental fit indices (Hu & Bentler 1999; Kline 2016; Little 2013; χ^2^/df ≤ 2[3]; RMSEA ≤ .06 [.08]; SRMR ≤ .08 [.10]; CFI ≥ .95 [.90]), and we used the robust maximum likelihood estimation method, MLR. To ensure the stability of the measurement model over time and across groups, we conducted nested model tests for measurement invariance. We started with a complex model, i.e., the model in which the same indicators are linked to the same constructs at both time points without any other constraints, and progressively added constraints, i.e., simplifying the model, monitoring model fit, and only continuing if the decrease in fit was not significant (Little 2013). For model comparisons, we used differences in scaled chi-square statistics and CFI (Comparative Fit Index) values. Significant drops in chi-square or ΔCFI > |.01| indicated a need to retain the more complex model or achieve partial invariance (Cheung & Rensvold 2002; Putnick & Bornstein 2016). We used the same analytical procedure to determine longitudinal multi-group invariance across ethnic and contextual groups at T1 and T2 (see Appendix D).

**Figure 1:**
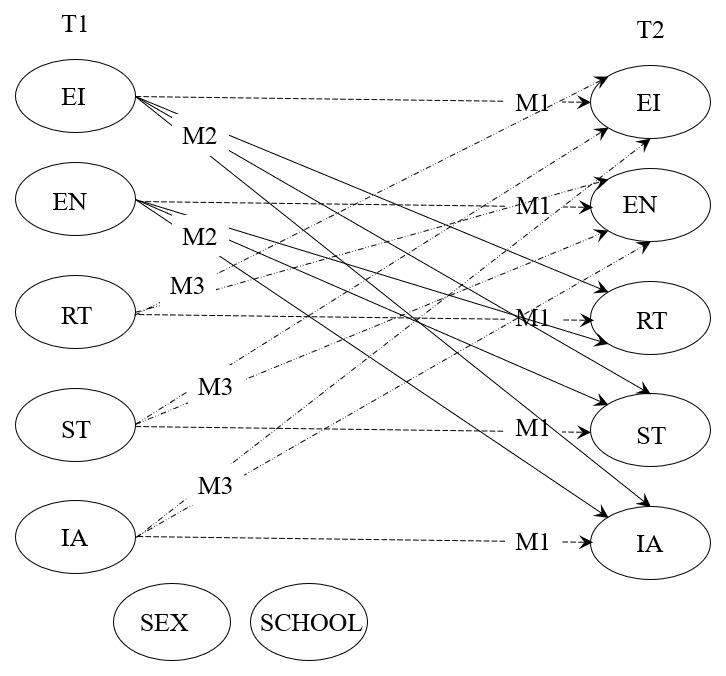
Structural AR-CLP model of longitudinal effects between ethnic identity and ethnonationalism (EI and EN), perception of realistic and symbolic intergroup threat (RT and ST), and perception of intergroup anxiety (IA). *Note*. All paths that we tested within the framework of four nested models are depicted. M1 (dotted lines) represent the reference stability model, M2 (solid lines) represent the assumed causality model, M3 (dashed lines) represent the reverse causality model, and finally, the full reciprocal causality model (M4) is obtained by including all the paths from M1 to M3. T1 = first measurement point, T2 = second measurement point. Circles represent constructs (latent variables). Paths from two covariates at T1, sex and school type (elementary or high school), to constructs at T2 are not depicted for clarity but are included in all analyses.

After confirming measurement models, we set up longitudinal autoregressive cross-lagged panel models (AR-CLPM) to test temporal relationships between constructs (see Figure 1).

AR-CLPM is appropriate since we have two measurement points in which data were collected, and we are primarily interested in the direction and strength of relationships between assumed predictors and criteria (Urbanaviciute, Udayar & Rossier 2019). The decision criterion for retaining a model was the difference in the size of the scaled chi-square (scaled ∆χ²). The reference model for comparing different nested models was the stability model, which is the model where we only specified autoregressive coefficients and correlations between variables measured at the same time point. To establish relationships between predictor and criterion variables, we tested the fit of: (1) the assumed causality model (adding paths from ethnic identity and ethnonationalism measured at T1 to perceived realistic and symbolic threat and intergroup anxiety measured at T2), (2) the reverse causality model (adding paths from perceived realistic and symbolic threat and intergroup anxiety measured at T1 to ethnic identity and ethnonationalism measured at T2), and (3) the reciprocal causation model with all cross-lagged paths. Analogous to the traditional regression approach, we also specified two assumed covariates as semi-partial control—participant sex and school type (elementary or high school). Covariates predict constructs only at the T2, while having a non-directive connection with constructs from the T1. The assumed moderation of status and context was tested using multigroup analysis, which is recommended when the moderator is a dichotomous variable (Little 2013).

## Results

Prior to testing our hypothesis, we analyzed missing data in our longitudinal sample and established that there was no issue with data bias (see Appendix E). To handle missing data, we used a model—based missing data method—FIML (full information maximum-likelihood).

### Preliminary Analyses

The arithmetic means and standard deviations are shown in Table 1.

**Table 1:** Descriptive statistics for the total sample, and separately for context and status for both time points.


MEASUREMENT POINT	VARIABLE		CONTEXT		STATUS	
	
		TOTAL SAMPLE *n* = 852	POST-CONFLICT *n* = 360	NON-CONFLICT *n* = 492	MAJORITY *n* = 485	MINORITY *n* = 367

		*M (SD)*	*M (SD)*	*M (SD)*	*M (SD)*	*M (SD)*

T1	Ethnic identity	4.13 (0.869)	4.28 (0.835)	4.02 (0.876)	4.14 (0.860)	4.12 (0.880)

	Ethnonationalism	2.43 (1.113)	2.57 (1.183)	2.33 (1.048)	2.43 (1.113)	2.43 (1.115)

	Realistic threat	2.50 (0.966)	2.74 (1.036)	2.32 (0.871)	2.39 (0.914)	2.64 (1.014)

	Symbolic threat	2.28 (1.061)	2.79 (1.045)	1.90 (0.904)	2.14 (1.022)	2.47 (1.083)

	Intergroup anxiety	1.80 (0.716)	1.94 (0.767)	1.70 (0.658)	1.78 (0.693)	1.82 (0.746)

T2	Ethnic identity	4.02 (0.939)	4.26 (0.872)	3.84 (0.948)	4.07 (0.943)	3.95 (0.930)

	Ethnonationalism	2.30 (1.121)	2.47 (1.152)	2.17 (1.082)	2.31 (1.153)	2.29 (1.079)

	Realistic threat	2.51 (1.029)	2.80 (1.051)	2.30 (0.961)	2.43 (1.013)	2.62 (1.041)

	Symbolic threat	2.19 (0.996)	2.61 (0.968)	1.88 (0.896)	2.05 (0.960)	2.37 (1.014)

	Intergroup anxiety	1.79(0.708)	1.89 (0.754)	1.72 (0.664)	1.79 (0.702)	1.79 (0.716)


*Note*. M and SD refer to manifest variables. T1 = first measurement point; T2 = second measurement point. The range of scores on all scales is from 1 to 5.

Ethnic identity is highly expressed among all participants (*M*_T1_ = 4.13; *M*_T2_ = 4.02), while ethnonationalism does not exceed the neutral point of the scale in any (sub)sample (*M*_T1_ = 2.43; *M*_T2_ = 2.30). Relatively low, i.e., below the neutral value of the scale, are also the results for perceived realistic (*M*_T1_ = 2.50; *M*_T2_ = 2.51) and symbolic (*M*_T1_ = 2.28; *M*_T2_ = 2.19) threat, as well as intergroup anxiety (*M*_T1_ = 1.80; *M*_T2_ = 1.79).

Pearson correlation coefficients between the total scores on the scales of ethnic identity, ethnonationalism, perceived realistic and symbolic intergroup threat, and intergroup anxiety across the entire sample (*N* = 852) at both time points are shown in Table 2. We observe that ethnic identity has a very low positive correlation only with perceived symbolic intergroup threat, while ethnonationalism is low to moderately highly positively correlated with all forms of intergroup threat.

**Table 2:** Intercorrelation of the research variables for both time points.


VARIABLE	1.	2.	3.	4.	5.

1. Ethnic identity	–	.40**	.01	.12**	.07

2. Ethnonationalism	.44**	–	.17**	.29**	.32**

3. Realistic threat	–.02	.18**	–	.41**	.20**

4. Symbolic threat	.07*	.23**	.48**	–	.48**

5. Intergroup anxiety	.001	.22**	.29**	.48**	–


Note. *p < .05; **p < .01. First measurement results (T1) below diagonal, second measurement results (T2) above diagonal.

### Longitudinal Effects of Ingroup Identification on the Perception of Intergroup Threat and Intergroup Anxiety

We analyzed how ingroup identification affects perceptions of intergroup threat and anxiety over time, using a measurement model with constraints from longitudinal measurement invariance tests. In Table 3, we displayed fit indices for all models tested, and Table 4 shows the unstandardized and standardized coefficients for stability and cross-lagged effects in our final, reciprocal causation, model. Results showed that constructs are stable over time, regardless of cross-lagged paths. Adding directional paths from identity variables at T1 to perception of intergroup threat and anxiety at T2 improved model fit. However, the only significant relationship was from ethnonationalism at T1 to symbolic intergroup threat at T2. The reverse causality model showed better fit, with significant paths from symbolic intergroup threat at T1 to ethnic identity at T2, and from intergroup anxiety at T1 to ethnonationalism at T2. Finally, Model 4, which included all significant effects from Models 2 and 3, had the best fit.

**Table 3:** Cross-lagged model fit and comparison of alternative models.


MODEL	SCALED χ^2^	df	*p*	RMSEA [90% CI]	SRMR	CFI	COMPARED MODELS	SCALED Δχ^2^	ΔDF	Δχ^2^ Cr.	*p*

0	17277.692	818	<.001	–	–	–	–	–	–	–	–

1	1299.762	686	<.001	0.034 [0.031; 0.037]	0.050	0.964	–	–	–	–	–

2	1281.934	680	<.001	0.034 [0.031; 0.037]	0.046	0.964	1–2	17.83	6	12.59	.007*

3	1260.171	680	<.001	0.033 [0.030; 0.036]	0.043	0.966	1–3	39.59	6	12.59	<.001*

*4*	*1244.530*	*674*	*<.001*	*0.033* *[0.030; 0.036]*	*0.040*	*0.966*	*1–4*	*55.23*	*12*	*21.03*	<.001*

							2–4	37.40	6	12.59	<.001*

							3–4	15.64	6	12.59	.02*


*Note*. The final model is in italic. Models: 0 = null; 1 = autoregressive; 2 = assumed CL paths; 3 = reverse CL paths; 4 = reciprocal causation. Scaled χ^2^ = value with Yuan–Bentler correction, Δ = difference in parameters between two nested models, Δχ^2^ Cr. = boundary value above which the difference between compared models is statistically significant at a *p* < .05 level for corresponding degrees of freedom (Δdf).

**Table 4:** Stability and cross-lagged effects in reciprocal causation AR-CLP Model (N = 852).


MODEL	AR PATH	B	β	CL PATH	B	β

Reciprocal causation	EI_T1_ → EI_T2_	0.703*	0.644*	EI_T1_ → RT_T2_	0.107	0.085

	EN_T1_ → EN_T2_	0.571*	0.564*	EI_T1_ → ST_T2_	0.012	0.009

	RT_T1_ → RT_T2_	0.745*	0.675*	EI_T1_ → IA_T2_	0.018	0.019

	ST_T1_ → ST_T2_	0.568*	0.611*	EN_T1_ → RT_T2_	–0.048	–0.056

	IA_T1_ → IA_T2_	0.518*	0.547*	*EN_T1_* → *ST_T2_*	0.092*	0.105*

				EN_T1_ → IA_T2_	0.064	0.100

				RT_T1_ → EI_T2_	–0.051	–0.053

				RT_T1_ → EN_T2_	0.003	0.002

				*ST_T1_* → *EI_T2_*	0.105*	0.135*

				ST_T1_ → EN_T2_	0.090	0.084

				IA_T1_ → EI_T2_	0.006	0.005

				*IA_T1_* → *EN_T2_*	0.240*	0.159*


*Note*. *Statistically significant path – 95% confidence interval does not include zero to two decimals. Effects of covariates were included in all analyses as controls. AR = autoregressive path (stability coefficient), B = unstandardized regression coefficient, β = standardized regression coefficient, CL = cross-lagged path, EI = ethnic identity, EN = ethnonationalism, RT = realistic intergroup threat, ST = symbolic intergroup threat, IA = intergroup anxiety. T1 = first measurement point, T2 = second measurement point.

Findings suggest a reciprocal relationship, i.e., more intense intergroup anxiety increases ethnonationalistic sentiments, while stronger ethnonationalistic sentiments predict a stronger perception of symbolic intergroup threat. Finally, stronger symbolic intergroup threat over time leads to greater ethnic attachment.

The predictors from the first time point explained the following variances at T2: 45.8% in ethnic identity, 46.7% in ethnonationalism, 47.7% in realistic intergroup threat perception, 44.6% in symbolic intergroup threat perception, and 35.2% in intergroup anxiety perception.

### Moderating Effects of Group Status (Majority/Minority) and Context (Post-Conflict/Non-Conflict)

To test the moderating effects of group status (majority/minority) and context (post-conflict/non-conflict) on relationships in the AR-CLP model (model 4), we repeated multi-group tests of nested models. The initial unconstrained multi-group model fit well for both group status, χ² (1356) = 2164.095, *p* < .001, χ²/df = 1.60, CFI = 0.955, RMSEA = 0.039 [0.036, 0.042], SRMR = 0.051, and context, χ² (1362) = 2108.308, *p* < .001, χ²/df = 1.55, CFI = 0.959, RMSEA = 0.037 [0.034, 0.040], SRMR = 0.049. The model with constrained cross-lagged paths did not show significantly worse fit for either group status, Δχ^2^(12) = 10.53, *p* = .638, or context, Δχ^2^(12) = 18.76, *p* = .066. These results suggest that AR-CLP model fits equally well for both majority and minority groups, and for both post-conflict and non-conflict contexts, indicating no significant moderating effects of group status or context.

## Discussion

This research aimed to explore how different forms of ingroup identification predict various forms of intergroup threat among adolescents in multiethnic communities in Croatia. We investigated whether these relationships were moderated by the majority–minority status of the group and the multiethnic context of the study. By examining both majority and minority groups, we were able to examine the equivalence of longitudinal effects between these groups, thereby identifying similarities and differences in the experiences of groups with different statuses. Additionally, our study addresses a gap in the literature by examining how the context moderates these relationships. Specifically, we assessed whether the relationships among variables of ingroup identification and perception of threat differ between groups from Vukovar, a post-conflict area where social recovery is ongoing, and non-conflict areas with long-standing multicultural integration. This context-specific research contributes valuable insights to the theory and practice of understanding and addressing intergroup relations.

### Longitudinal Effects of Ingroup Identification on the Perception of Intergroup Threat and Intergroup Anxiety

Participants in this study reported strong ingroup attachment, expressing ethnic pride without negative tendencies toward other ethnic groups. Ethnonationalist ideology was also present, marked by the belief in the superiority and moral righteousness of one’s nation. However, the average results did not surpass the neutral point on the scale, similar to outcomes observed with various operationalizations of intergroup threat, suggesting that national minorities were not perceived as a significant threat to the majority group, nor vice versa. This can be explained by the fact that these minorities exercise their rights, but they are not threatening as they are numerically much smaller than Croats.

Our data best fit the reciprocal model, showing three significant positive cross-effects: the effect of intergroup anxiety on ethnonationalism, the effect of ethnonationalism on symbolic threat, and the effect of symbolic threat on ethnic identity.

This study differs from typical intergroup threat research on (im)migrants or asylum seekers, as it involves groups that have coexisted for many years, allowing threats to develop from existing attitudes and interactions. Given that the participants in the study are adolescents, it is possible that they are under the influence of transgenerational transmission of intergroup attitudes and norms (Stasiuk & Bilewicz 2013). Having already developed some perception of the other group as more or less threatening, the idea of coming into contact with members of that group can result in anxiety. Authors of ITT emphasize the reciprocity of relationships among variables in the model, and as we only had two measurement points in this study, we cannot conclude about the further relationship among variables (Stephan & Stephan 2018). However, we can assume that at some point, ingroup attachment operationalized through social identities of ethnic belonging and ethnonationalism could predict the perception of threat. This thinking is supported by our finding confirming that ethnonationalism, emphasizing group superiority, makes individuals more sensitive to symbolic threat and protective of their identity. Individuals with strong ingroup attachment perceive greater threats, as their group membership is a crucial part of their self-concept (Riek, Mania & Gaertner 2006). Their group membership serves as a lens through which they view relationships in their social context (Uzelac et al. 2022; Verkuyten 2009) where they view their group as a moral compass, seeing others as threats to their values and traditions. This sensitivity to symbolic threat leads to the protection of their identity by emphasizing their group’s importance. Our findings align with research showing that nationalism, through symbolic threat, can lead to open prejudices toward Roma among adolescents (Dimitrova et al. 2015; Ljujic, Vedder & Dekker 2012). Skokandić (2018) also confirms the role of nationalism in perceiving symbolic threats from traditional ethnic minorities in Croatia. Our research expands these findings to show that they are equally applicable to traditional ethnic minorities who have lived as minorities in Croatian territories for many decades and centuries, as well as to the new Serbian minority, whose status changed from one of the constituent nations in the former Republic of Yugoslavia to minority group after the Homeland War of 1991–1995.

The prominent and stable effect of intergroup anxiety is understandable given the sample and context. Adolescents are particularly sensitive to peer influence and concerned about their social image (Nekić, Uzelac & Jurkin 2016). In schools, where peer dynamics are intense, intergroup anxiety—a type of affective and interpersonal threat—is especially impactful. Strengthening ethnonationalism might be adolescents’ way of coping with unpleasant emotions and reinforcing a sense of belonging that is threatened by negative interactions or their anticipation. This is in line with the rejection-identification model (Branscombe et al. 1999b) which posits that perceived ingroup discrimination (i.e., threat) may lead to increased identification with one’s group, to maintain well-being. Indeed, previous research shows that unpleasant emotions toward outgroups, such as anger, enhance ingroup identification (Kessler & Hollbach 2005) and make group membership a salient identity during subsequent encounters. Similarly, our study indicates that intergroup anxiety leads to increased ethnonationalism. Adolescents may rationalize their anxiety by believing that only their group is correct, while others are less valuable and less trustworthy, thus reconciling their feelings and thoughts.

In addition to this affective type of threat, the perception of a more cognitive, symbolic threat also leads to stronger ingroup identification in form of an ethic identity. Given that symbolic threat arises when another group is perceived as endangering to the identity and worldview of one’s own group, it is plausible that strengthening ingroup attachment would serve as a protection of threatened identity. In our study, both intergroup anxiety and symbolic threat impact identity more than perceived realistic threat. Strengthening ingroup ties may not address realistic threats; instead, adolescents might avoid close contact with the threatening group. However, stronger ingroup identification can mitigate the negative effects of intergroup anxiety and symbolic threat, protecting psychological well-being. This aligns with the threat-identification model (Schmid & Muldoon 2015), which suggests that perceived threats can indirectly enhance well-being through stronger ingroup identification. Thus, stronger ingroup identification can be a coping strategy for perceived threats coming from media, political authorities, or ingroup stereotypes and prejudices about the outgroup.

### Moderating Effects of Group Status (Majority/Minority) and Context (Post-Conflict/Non-Conflict)

Several factors may explain why we failed to confirm the hypothesized moderating effects. On the one hand, it is possible that moderating effects of group status and multiethnic context in the hypothesized relationships may be very weak, rendering them insignificant for the relationship between ingroup identification and intergroup threat experience; however, on the other hand, there are some methodological reasons which should be mentioned. Although our sample size was sufficient to detect medium effect sizes (Moshagen & Erdfelder 2016), increasing the sample size could enhance the precision of parameter estimates, potentially making small effects statistically significant. Additionally, analyses of moderating effects, like all analyses within SEM, depend on the validity and reliability of measured instruments and the specification of the model itself. More research and different analytical approaches are necessary to firmly establish these relationships. Along those lines, although our two-item measure captured the core of ethnonationalism, that is, the idealization and glorification of one’s own ethnic group, we are aware of the limitations associated with such a narrow operationalization. We therefore recommend that future research use a more extensive operationalization with more nuanced items to capture additional aspects of the construct, including various ways of expected ingroup privilege and outgroup derogation. A more elaborated measure of ethnonationalism might show stronger associations with threat perception and weaker associations with ethnic identity, which would further contribute to distinguishing these constructs both conceptually and practically. Moreover, when two moderators are closely related, as in our case with context and status, it would be preferable to test the effects of both moderators simultaneously on a sufficiently large sample. This would provide insights into the specifics of majority–minority dynamics within a specific context, as well as groups of equal status across different contexts. This limitation of our study represents a promising vein for future research.

It is important to note that a lack of statistical evidence for moderation does not imply that the constructs do not interact in reality. Psychological measures may not perfectly represent the underlying constructs, meaning established relationships among measures do not exactly replicate the relationships among the actual phenomena.

Our results indicated contextual equivalence in the significant relationships between ingroup identification and the perception of intergroup anxiety and symbolic threat. The statistically equivalent longitudinal relationships suggest that the variables interact similarly across different groups, regardless of status and context, thus supporting the generalization of our findings. This would mean that the processes linking ingroup identification and threat perception in our sample are less sensitive to broad historical and status distinctions, making ITT assumptions equally applicable. The absence of the expected moderation may also be explained by characteristics of our sample, which consists of adolescents without direct experience of conflict, with the study conducted roughly two decades after the war. It seems that adolescents in our post-conflict context do not perceive the consequences of the conflict as significant determinants of their everyday experiences, and that threat perception is an equally relevant factor in intergroup relations as in non-conflict contexts or those with an even more distant history of conflict. Furthermore, our findings suggest that minority adolescents resemble their majority peers in their intergroup cognition. The developmental stage may override status differences because identity exploration, peer norms, and school environments may exert greater and relatively uniform influence on intergroup attitudes. This suggests that adolescents from multiethnic contexts resemble one another because they have not yet internalized broader social norms to the same extent as adults (Löw Stanić 2014). They may also never fully adopt these norms but instead reshape them. Majority–minority differences in threat experiences and reactions may emerge later in life if political socialization, structural inequalities, and lived experiences begin to diverge. The applicability of ITT among participants from different contexts, generations, and developmental stages would certainly represent a compelling area for further research. As for this study and these findings, we find them encouraging for designing interventions aimed at fostering closer relations among different ethnic groups.

### Contributions and Practical Implications

In multiethnic contexts, groups tend to be very conscious of their differences and ethnic identity is very salient. However, this does not imply high ethnonationalism nor perception of the outgroup as a threat. Not even in a post-conflict context, which is very promising finding. Nevertheless, even in such circumstances, perceiving an outgroup as a threat and feeling anxious around the outgroup members leads to more ethnonationalism, retreating into the boundaries of ingroup and appreciating only what is familiar. In the long run, that intergroup distancing can have detrimental effects for all members of multiethnic community, regardless of status. Thus, narratives that portray the outgroup as threatening are dangerous for intergroup relations, just as it is undesirable to build a social identity around glorifying one’s own group at the expense of others.

Our results highlight the complexity of relationships between identity variables and the perception of threat, suggesting several directions for action. Educating about diversity can reduce intergroup anxiety and, consequently, ethnonationalist sentiments. Embracing and promoting diversity as a valuable asset to the community can diminish the exclusive attachment to one’s own group based on comparison and competition (ethnonationalism). Celebrating ethnic diversity through various traditions and cultures at schools and within local communities can foster a common identity, where each group contributes to a shared sense of belonging.

By emphasizing how each ethnic group enriches a common, inclusive identity (e.g., town, regional) rather than which group is superior, the experience of symbolic threat from outgroups can be reduced. This reduction in perceived symbolic threat will also lessen strong ethnic identification, which, in our contexts, is beneficial. Ethnic divisions, often perpetuated by separate schooling systems, hinder intergroup interaction and understanding (Čorkalo Biruški & Ajduković 2007, 2008a, 2008b, 2012). Of course, we are aware that it is necessary to enable ethnic specificities because, as our results show, it is not ethnic identity but ethnonationalism that leads to symbolic threat and deteriorates intergroup relations (Jelić, Čorkalo Biruški & Rebernjak 2022). Therefore, it is crucial to address the community’s needs and interests, such as promoting bilingualism in majority and minority languages, only if it does not provoke a perceived threat from the majority group. Otherwise, the effects could be just the opposite of the desired ones. Intergroup rapprochement should be given time and proceed at a pace that will be acceptable to the majority, while still ensuring that the minority sees there is openness to diversity and respect for their identity.

Peers are vital for the development of social identities (Umaña-Taylor et al. 2020), making the school environment a key place to influence adolescents positively. In ethnically homogeneous schools, teachers play a crucial role in transmitting desirable norms about intergroup relations. Addressing intergroup relations with adolescents is challenging and requires understanding their existing attitudes and experiences. However, adolescents can handle complex messages about intergroup similarities, differences, and historical contexts, making it necessary to engage with them on these sensitive issues.

## Conclusion

We partially confirmed one of our hypotheses by showing that participants with higher levels of ethnonationalism at T1 experienced higher levels of symbolic threat at T2. Additionally, we found that increased symbolic intergroup threat over time led to greater attachment to one’s ethnic group, and that heightened intergroup anxiety over time increased ethnonationalistic sentiments. However, we did not confirm the hypothesized moderating effects of majority–minority status and post-conflict versus non-conflict contexts. The finding of statistically equivalent longitudinal relationships among variables suggests that these relationships function similarly across different groups, regardless of status and context, thereby supporting the generalization of our findings.

In conclusion, this research provides valuable insights into the intergroup relations of adolescents in multiethnic environments and the role of threat perception. However, to better capture the dynamics of intergroup variables, future research should include multiple measurement points over a longer period. We hope this paper will inspire new, methodologically robust research that offers a more comprehensive understanding of the risk factors for intergroup threat and its consequences. We also hope our recommendations will encourage initiatives aimed at fostering connections among children and adolescents.

## Additional File

The additional file for this article can be found as follows:

10.5334/irsp.983.s1 Supplementary Online Material. Appendixes A to E.

## Footnotes

See Appendix C for full list of items.

Although the term nation is used in the items, in line with previous studies, in the Croatian language it has the same meaning as an ethnic group and is different from identification with the state.
