# Supplementary material for: Understanding Ingroup Identification and Intergroup Threat Dynamics in Multiethnic Contexts
Source: Int Rev Soc Psychol. 2026 Mar 23;39:18. doi: 10.5334/irsp.983 (PMC13025153; doi:10.5334/irsp.983)
Supplement: Supplementary Online Material. — Appendixes A to E. [file irsp-39-983-s1.pdf]

Supplementary Online Material

Appendix A

Structure of participants by ethnicity in the targeted sampling locations, based on the 2011 census

Context	Research site	Total population	Croats	The first most numerous minority	The second most numerous minority	The third most numerous minority
Croatian-Serbian	Vukovar	27 683	15 881 (57.4%)	*Serbs: 9 654 (34.9%)*	Rusyns: 440 (1.6%)	Hungarians: 347 (1.3%)
	Dalj	3 937	1 474 (37.4%)	*Serbs: 2 163 (55%)*	Hungarians: 213 (5.4%)	Germans: 12 (0.3%)
	Tenja	7 376	5 251 (71.2%)	*Serbs: 1 903 (25.8%)*	Hungarians: 46 (0.6%)	Roma: 37 (0.5%)
	Jagodnjak	2 023	391 (19.3%)	*Serbs: 1 333 (65.9%)*	Roma: 154 (7.6%)	Hungarians: 61 (3%)
	Mirkovci	3 283	2 031 (61.9%)	*Serbs: 1 165 (35.5%)*	Rusyns: 15 (0.5%)	-
Croatian-Hungarian	Osijek	108 048	96 746 (89.5%)	Serbs: 6 751 (6.3%)	*Hungarians: 979 (0.9%)*	Albanians: 437 (0.4%)
	Zmajevac	853	271 (31.8%)	*Hungarians: 546 (64%)*	Serbs: 21 (2.5%)	-
	Korođ (Korog)	485	136 (28%)	*Hungarians: 343 (70.7%)*	-	-
	Novi Bezdan	300	48 (16%)	*Hungarians: 243 (81%)*	-	-

	Lug	764	152 (19.9%)	*Hungarians: 565 (74%)*	Serbs: 19 (2.5%)	Germans: 16 (2.1%)
	Tordinci	2 032	1 577 (77.6%)	*Hungarians: 371 (18.3%)*	Serbs: 72 (3.5%)	-
Croatian-Czech	Daruvar	11 633	7 129 (61.3%)	*Czechs:* 2 485 (21.4%)	Serbs: 1 429 (12.3%)	Hungarians: 114 (1%)
	Končanica	2 360	983 (41.7%)	*Czechs:* 1 110 (47%)	Serbs: 180 (7.6%)	Hungarians: 43 (1.8%)
	Dežanovac	2 715	1 598 (58.9%)	*Czechs:* 627 (23.1%)	Serbs: 318 (11.7%)	Hungarians: 111 (4.1%)
Croatian-Italian	Rovinj	14 294	9 054 (63.3%)	*Italians:* 1 608 (11.2%)	Serbs: 594 (4.2%)	Bosnians: 295 (2.1%)
	Buje	5 182	2 525 (48.7%)	*Italians:* 1 261 (24.3%)	Serbs: 181 (3.5%)	Slovenians: 170 (3.3%)
	Umag	13 467	8 128 (60.4%)	*Italians:* 1 962 (14.6%)	Serbs: 590 (4.4%)	Bosnians: 464 (3.5%)
	Vodnjan	6 119	3 160 (51.6%)	*Italians:* 1 017 (16.6%)	Bosnians: 425 (7%)	Roma: 299 (4.9%)

Note. Target minority is in italic.

Appendix B

Structure of participants with regard to the school and class they attended at the first and second measurement points

Measurement point	Context	Type of school based on the language of instruction	1st grade of high school	2nd grade of high school	3rd grade of high school	4th grade of high school	7th grade of elementary school	8th grade of elementary school	Total
T1	Post-conflict	Majority	-	-	-	-	26	-	26
		Mixed	88	91	88	-	67	-	334
		Total	88	91	88	-	93	-	360
	Non-conflict	Majority	2	68	63	-	83	-	216
		Minority	45	31	43	-	57	-	176
		Mixed	4	20	22	-	51	3	100
		Total	51	119	128	-	191	3	492
	Total	Majority	2	68	63	-	109	-	242
		Minority	45	31	43	-	57	-	176
		Mixed	92	111	110	-	118	3	434
		Total	139 (16.3%)	210 (24.6%)	216 (25.4%)	-	284 (33.3%)	3 (0.4%)	852 (100%)
Measurement	Context	Type of school based	1st grade of high school	2nd grade of	3rd grade of high school	4th grade of high school	7th grade of elementary	8th grade of elementary	Total

point		on the language of instruction		high school			school	school	
T2	Post-conflict	Majority	-	-	-	-	-	26	26
		Mixed	-	88	91	88	1	66	334
		Total	-	88	91	88	1	92	360
	Non-conflict	Majority	-	2	69	62	-	83	216
		Minority	5	43	32	42	-	57	179
		Mixed	-	4	20	22	-	51	97
		Total	5	49	121	126	-	191	492
	Total	Majority	-	2	69	62	-	109	242
		Minority	5	43	32	42	-	57	179
		Mixed	-	92	111	110	1	117	431
		Total	5 (0.6%)	137 (16.1%)	212 (24.9%)	214 (25.1%)	1 (0.1%)	283 (33.2%)	852 (100%)

Note. N=1 is repeating 7th grade, N=2 is repeating 1st grade, N=1 is repeating 2nd grade, N=2 is repeating 3rd grade.

Appendix C

Survey questionnaire items

Ethnic identity scale

A five-point scale ranging from strongly disagree (1) to strongly agree (5).

1. I am glad to be a member of my nation.

2. I feel strong ties with members of my nation.

3. Belonging to my nation is very important to me.

4. I feel that I belong to my nation.

5. Belonging to my nation makes me proud.

Ethnonationalism scale items

A five-point scale ranging from strongly disagree (1) to strongly agree (5).

1. My nation is better than other nations.

2. I would rather belong to my nation than any other nation.

3. In all historical conflicts with other nations my nation was always right.

Perceived realistic threat scale items

A five-point scale ranging from strongly disagree (1) to strongly agree (5).

1. It is more likely that a job in our town will get [out-group member] than [in-group member].

2. [Out-group members] in our town have more privileges (rights) than anywhere else.

3. Students whose language of instruction is [out-group] have more opportunities to participate in school competitions than students whose language of instruction is [in-group].

4. Students whose language of instruction is [out-group] have more opportunities to go on field trips and school trips than students whose language of instruction is [in-group].

5. Students whose language of instruction is [out-group] are given more concessions than students whose language of instruction is [in-group].

Perceived symbolic threat scale items

A five-point scale ranging from strongly disagree (1) to strongly agree (5).

1. My [out-group] peers do not respect the language of my nation.

2. My [out-group] peers should not overemphasize their national symbols and customs.

3. My [out-group] peers are listening the music that bothers us.

4. My [out-group] peers think they are better than we are.

5. My [out-group] peers are name-calling us because we are of different nationality.

Intergroup anxiety

A five-point scale ranging from not at all (1) to extremely (5). It consisted of six items that ask participants how they would feel when interacting with members of the other ethnic group (e.g. having a conversation with them, collaborating on a school task, etc.).

1. Comfortable ^{R}

2. Nervous

3. Friendly ^{R}

4. Uncertain

5. Worried

6. Equal ^{R}

Note. Items in italics have been excluded from further analyses based on the results of factor analyses.

Appendix D

Measurement invariance analyses.

All constructs in the table are latent variables. The final models are in bold. EI/EN = ethnic identity and ethnonationalism, two factors specified in the identity model. RT/ST = perception of realistic and symbolic threat, two factors specified in the threat model. IA = perception of intergroup anxiety. Scaled $\chi2$ = value with Yuan-Bentler correction, Δ = difference in parameters between two nested models, $\Delta\chi2$ Cr. = critical value above which the difference between compared models is statistically significant at the p<.05 level for the corresponding degrees of freedom (Δdf), the critical value above which the difference between compared models is statistically significant for ΔCFI is always the same and is .01. All values of model fit indices represent robust estimates.

Table C1

Longitudinal measurement invariance analyses (N=852)

Construct	Model	Scaled $\chi2$	df	p	Scaled $\chi2$ /df	Δdf	$\Delta\chi2$	$\Delta\chi2$ Cr.	RMSEA [90% CI]	SRMR	CFI	ΔCFI
EI/EN	Null	6449.718	105	<.001	-	-	-	-	-	-	-	-
	Configural	160.537	64	<.001	2.51	-	-	-	0.046 [0.037; 0.055]	0.026	0.985	-
	**Metric**	**170.088**	**69**	**<.001**	**2.48**	**5**	**9.55**	**11.07**	**0.045 [0.037; 0.054]**	**0.029**	**0.985**	**-0.001**

RT/ST	Null	5976.236	171	<.001	-	-	-	-	-	-	-	-
	Configural	360.756	116	<.001	3.11	-	-	-	0.054 [0.048; 0.060]	0.047	0.958	-
	**Metric**	**373.735**	**123**	**<.001**	**3.04**	**7**	**12.979**	**14.067**	**0.053 [0.047; 0.059]**	**0.049**	**0.957**	**-0.001**
IA	Null	2021.713	21	<.001	-	-	-	-	-	-	-	-
	Configural	6.679	5	0.246	1.34	-	-	-	0.022 [0.000; 0.060]	0.012	0.999	-
	**Partial metric**	**7.060**	**6**	**0.315**	**1.18**	**1**	**0.381**	**3.84**	**0.016 [0.000; 0.055]**	**0.013**	**1.000**	**0**

Table C2

Longitudinal multi-group measurement invariance analyses for ethnic majority (N=485) and minorities (N=367)

Construct	Model	Scaled χ2	df	p	Scaled χ2 /df	Δdf	Δχ2	Δχ2 Cr.	RMSEA [90% CI]	SRMR	CFI	ΔCFI
EI/EN	Null	6794.890	224	<0.001	-	-	-	-	-	-	-	-
	Configural	244.748	128	<0.001	1.91	-	-	-	0.050 [0.040; 0.059]	0.032	0.983	-
	**Metric**	**269.240**	**143**	**<0.001**	**1.88**	**15**	**24.49**	**25**	**0.049 [0.040; 0.058]**	**0.037**	**0.982**	**-0.001**
RT/ST	Null	6555.894	360	<.001	-	-	-	-	-	-	-	-
	Configural	522.359	232	<.001	2.25	-	-	-	0.059 [0.052; 0.065]	0.058	0.954	-
	**Partial metric**	**541.405**	**245**	**<.001**	**2.21**	**13**	**19.046**	**22.362**	**0.058 [0.051; 0.064]**	**0.060**	**0.953**	**-0.001**

IA	Null	2099.317	48	<.001	-	-	-	-	-	-	-	-
	Configural	20.896	10	0.022	2.09	-	-	-	0.054 [0.020; 0.087]	0.021	0.995	-
	**Metric**	**31.133**	**16**	**0.013**	**1.95**	**6**	**10.24**	**12.59**	**0.052 [0.023; 0.079]**	**0.037**	**0.993**	**-0.002**

Table C3

Longitudinal multi-group measurement invariance analyses for post-conflict (N=360) and non-conflict context (N=492)

Construct	Model	Scaled χ2	df	p	Scaled χ2 /df	Δdf	Δχ2	Δχ2 Cr.	RMSEA [90% CI]	SRMR	CFI	ΔCFI
EI/EN	Null	6679.733	224	<0.001	-	-	-	-	-	-	-	-
	Configural	224.494	128	<0.001	1.75	-	-	-	0.046 [0.036; 0.055]	0.029	0.986	-
	**Metric**	**269.302**	**143**	**<0.001**	**1.88**	**15**	**44.81**	**25**	**0.050 [0.040; 0.059]**	**0.047**	**0.982**	**0.004**
RT/ST	Null	6635.851	360	<.001	-	-	-	-	-	-	-	-
	Configural	474.669	232	<.001	2.05	-	-	-	0.053 [0.046; 0.060]	0.050	0.962	-
	**Metric**	**501.470**	**253**	**<.001**	**1.98**	**21**	**26.801**	**32.671**	**0.052 [0.045; 0.058]**	**0.053**	**0.961**	**-0.001**
IA	Null	2100.956	48	<.001	-	-	-	-	-	-	-	-
	Configural	15.025	10	0.131	1.50	-	-	-	0.038 [0.000; 0.075]	0.017	0.998	-
	Partial metric	23.209	14	0.057	1.66	4	8.18	9.49	0.043 [0.007; 0.064]	0.028	0.996	-0.002

Appendix E

To address potential bias from systematic data attrition in longitudinal studies, we compared the results of participants with data from both time points (*N* = 852) to those with data only from the first time point (*N* = 183) on the variables of interest. We conducted four chi-square tests to check if participants tracked longitudinally differed from those from the T1 in terms of sex, living standard, context, and ethnic status. The only significant difference was that the longitudinal sample had more female and fewer male participants (χ^2 (1) = 4.81; *p* = .03). However, the structure of participants in terms of context and status, the main moderating variables in the study, did not change (context: χ^2 (1) = 3.42; *p* = .06; status: χ^2 (1) = 0.27; *p* = .60). Additionally, participants who remained in the study did not differ from those who dropped out in terms of family living standards (χ^2 (4) = 5.26; *p* = .26).

We also checked for differences in the overall results of all predictor and criterion variables by conducting a one-way multivariate analysis of variance (MANOVA) between participants who remained in the study and those who dropped out, finding no differences in this case either (Wilk's Λ = .10, *F* (7, 898) = 0.58, *p* = .77).

Finally, we conducted two independent samples t-tests to see if participants who remained in the study differed from those who dropped out in terms of how distressing they found participation and how useful they found the study, finding no differences (distress: *t* (238.05) = 1.89; *p* = .06; usefulness: *t* (933) = -0.95; *p* = .34). Additionally, the risk of participation being distressing due to the study's topic was minimized, as indicated by the mean distress and usefulness ratings on a scale from 1 (not at all) to 5 (very much), ranging from *M* = 1.63 to *M* = 1.85 for distress ratings, and from *M* = 3.56 to *M* = 4.19 for usefulness ratings.

The percentage of missing data at the level of continuous items is very low, ranging from 0.2% to 3.4% at T1, and from 0.1% to 2.9% at T2. Further analysis of missing data

using the *md.pattern* function from the R package mice revealed no systematic pattern of nonresponse on items, with participants most often skipping one or two items. We have complete data for 709 participants at T1, 775 participants at T2, and 655 (77%) participants who participated at both time points.
